# Comparative proteomics reveals protein signatures shared between and unique to bona fide plant EVs and other plant-derived vesicles

**DOI:** 10.1093/plphys/kiag141

**Published:** 2026-03-31

**Authors:** Michele Bifolco, Elisa Cappetta, Alfredo Ambrosone

**Affiliations:** Department of Pharmacy, University of Salerno, Fisciano 84084, Italy; Department of Pharmacy, University of Salerno, Fisciano 84084, Italy; Department of Pharmacy, University of Salerno, Fisciano 84084, Italy

## Abstract

Extracellular vesicles are membrane-bound particles that mediate intercellular communication by transporting bioactive molecules. In plants, extracellular vesicles are essential for defence responses, cell wall remodeling, and interkingdom interactions, holding significant promise in agriculture, biotechnology, and nanomedicine. However, the characterization of plant extracellular vesicles, including their nomenclature and biological function, remains challenging. Nondestructive isolation methods typically yield more *bona fide* extracellular vesicles (genuine EVs or gEVs), while destructive approaches often result in a heterogeneous mixture of intracellular, extracellular, and artificial vesicles, collectively termed plant-derived vesicles, along with co-purifying contaminants released from disrupted cells. In this context, the identification and characterization of conserved proteins associated with extracellular vesicles, and the distinction of potential nonvesicular contaminants, are needed to provide critical insights into their biological roles and the functional significance of extracellular vesicles. Herein, we conducted an in silico comparative analysis using previously published proteomic datasets from gEVs and plant-derived vesicles. Using phylogenetic orthology inference and protein domain analysis, we identified conserved and distinct molecular features differentiating gEVs from plant-derived vesicles, alongside proteins likely co-purified as contaminants. Signal peptide prediction, transmembrane domain, and motif analyses were performed to uncover specific traits of genuine EV and plant-derived vesicle protein cargo. Our findings reveal substantial variability among plant-derived vesicles and identify evolutionarily conserved functions and molecular features specifically associated with genuine extracellular vesicles. Furthermore, our analyses shed light on potential secretion pathways of different classes of extracellular vesicle-associated proteins, as well as on their transport modes, likely distinguishing between luminal and membrane-associated cargo. Collectively, these results pave the way for a more comprehensive understanding of plant extracellular vesicle biology.

## Teaser

Identifying conserved molecular signatures that distinguish plant extracellular vesicles from heterogeneous plant-derived vesicles clarifies their identity and improves understanding of their biogenesis and functional roles.

## Introduction

Extracellular vesicles (EVs) are small membrane-bound, non-replicating particles released by cells that play critical roles in many biological processes, acting as key mediators of intercellular communication by transporting proteins, lipids, RNAs, and other bioactive molecules (Welsh et al. 2024).

In recent years, significant efforts have been devoted to identifying specific EV markers and typically EV-associated proteins to explore their diagnostic potential and elucidate the biological relevance of EV subpopulations in mammalian systems (Ciferri et al. 2021).

In plants, EVs are increasingly recognized for their potential applications in agriculture, biotechnology, and nanomedicine, due to their capacity to transport bioactive compounds (Lo et al. 2024). While their physiological role remains to be fully defined, plant EVs have been implicated in important processes such as defence responses, cell wall remodeling, and interkingdom communication with fungi (de la Canal and Pinedo 2018; Stotz et al. 2022). Moreover, growing evidence shows that plant EVs have beneficial effects on human health (Yu et al. 2020; Alfieri et al. 2021) such as anti-inflammatory, antitumoral, and antioxidant effects in vitro and in vivo (Fan et al. 2022; Fang and Liu 2022; Karamanidou and Tsouknidas 2022; Nemati et al. 2022; Tan et al. 2022; Wang et al. 2023).

It is important to note that, as in other organisms, the term EV in plants serves as an umbrella term encompassing various vesicle types, including exosomes, nanovesicles, microvesicles, and apoptotic bodies, each differing in size, biogenesis, and molecular cargo (Pinedo et al. 2021). A growing body of evidence indicates that plant EVs can be further classified into distinct subpopulations based on their cellular origin and molecular characteristics. For example, EVs isolated from the apoplast, the extracellular space between plant cells, are considered genuinely extracellular, as they are secreted naturally without compromising cell integrity. Within them, apoplastic EVs, typically collected by vacuum infiltration of plant organs, often carry specific protein markers (eg TET8, PEN1), as reported in the model species *Arabidopsis* (Rutter and Innes 2017; Koch et al. 2025). In addition, EVs recovered from hydroponic cultures or conditioned media of in vitro plant systems are similarly regarded as extracellular origin. Interestingly, EVs isolated from plant in vitro cultures closely resemble apoplastic EVs in both morphology and protein biocargo (De Palma et al. 2020; Woith et al. 2021; Boccia et al. 2022; Ambrosone et al. 2023; Vestuto et al. 2024). Consequently, plant in vitro cultures represent valuable biotechnological platforms for the study of EV populations.

The scenario becomes more complex when vesicles are isolated using destructive procedures, such as extraction from plant tissues like fruits, leaves, roots, or rhizomes. These methods often yield highly heterogeneous preparations containing not only authentic EVs but also vesicles of uncertain origin. Such vesicles may derive from intracellular compartments or result from the reorganization of cellular material following tissue disruption, leading to a mixed population in the final isolate. Moreover, these preparations are frequently enriched with nonvesicular particles, including ribonucleoprotein complexes and cellular debris released during cell lysis (Pocsfalvi et al. 2018). The presence of such contaminants complicates the characterization of plant EVs by interfering with the accurate identification and functional analysis of genuine extracellular vesicles. Preparations of this nature have been broadly referred to as exosome-like nanoparticles (ELNPs), plant-derived nanoparticles (PDNPs), plant-derived edible nanoparticles (PDENs), or, more generally, as plant-derived nano- and microvesicles.

This complexity reflects the heterogeneity observed in plant EVs as reported by current proteomic studies and underscores the need to identify core proteins and potential candidate markers of *bona fide* extracellular vesicles (de la Canal and Pinedo 2018; De Palma et al. 2020; Pinedo et al. 2021; Lian et al. 2022; Ambrosone et al. 2023; Huang et al. 2025; Rodríguez de Lope et al. 2025). It also highlights the importance of characterizing proteins shared with other types of nano- and microvesicles, as well as with nonvesicular particles or vesicles of uncertain origin. Such efforts are essential to ensure the accuracy, consistency, and reproducibility of plant EV research, particularly in the context of plant physiology and biotechnological applications.

In this study, we hypothesize that plant gEVs possess a distinct and evolutionarily conserved set of core proteins that define their biogenesis and functional specialization, distinguishing them from plant nano and microvesicles vesicles purified through destructive isolation methods (here referred to as plant-derived vesicles, PDVs). To test this hypothesis, we performed a comparative analysis of plant EV proteomes aimed at identifying and distinguishing proteins associated with genuine EVs (gEVs) from those likely representing contaminants or PDV-associated components. Our approach combined comparative bioinformatics analyses of protein conservation and functional domain architecture with in silico predictions of transmembrane regions and signal peptides, as well as sequence motif analyses to explore conserved functional elements in EV-associated proteins. By integrating these analyses, this work contributes to a more comprehensive profiling of gEVs and PDVs, enhancing our understanding of their molecular diversity and biological significance.

## Results

### Orthogroup identification and protein domain profiling in plant EV proteomes

A comprehensive comparative analysis of proteomic datasets from selected studies on plant gEVs (see Table 1) and PDVs (see Table 2) was conducted. To identify protein clusters, we performed an orthology inference analysis using OrthoFinder. Based on a total of 13,832 protein sequences, 2,490 orthologous groups (orthogroups, OGs) were identified. Of these, 1,639 were multispecies OGs containing closely related proteins across two or more species (ie well-defined OGs according to OrthoFinder's clustering). The complete lists of OGs and all associated proteins are provided in Supplementary Dataset S1. The remaining proteins, which did not cluster into multispecies OGs (eg singletons or species-specific groups), were subjected to further classification, generating additional OGs that were not included in the core analysis (Supplementary Dataset S2).

**Table 1:** List of studies used for proteomic data extraction related to gEVs.

Plant species	Protein number	EVs sampling	Isolation	Reference
*Helianthus annuus L.*	228	Vacuum infiltration	dUC	Regente et al. (2017)
*Actinidia chinensis*	2,749	Pollen collection	dUC	Suanno et al. (2023)
*S. lycopersicum* L.	192	Conditioned media collection	dUC	De Palma et al. (2020)
*A. thaliana/N. benthamiana*	1,936	Vacuum infiltration	dUC	He et al. (2021)
*Salvia dominica*	130	Conditioned media collection	dUC	Boccia et al. (2022)
*Salvia sclarea*	129	Conditioned media collection	dUC	Vestuto et al. (2024)
*Sorghum bicolor*	422	Vacuum infiltration	IDG-UC	Chaya et al. (2024)
*Arabidopsis thaliana*	466	Vacuum infiltration	IDG-UC	Rutter and Innes (2017)
*Cynara cardunculus*	87	Conditioned media collection	SEC	dataset identifier PXD059086

Abbreviations: differential ultracentrifugation (dUC); IDG-UC: iodixanol density gradient- ultracentrifugation.

**Table 2:** List of studies used for proteomic data extraction related to PDVs.

Plant species	Protein number	Isolation	Source	Reference
*Citrus sinensis L.* *Citrus limon L.* *Citrus paradisi L.* *Citrus aurantium L.*	(660 + 673 + 800 + 827) = 2,960	dUC	Fruit	Pocsfalvi et al. (2019)
*Citrus lanatus L.*	17	dUC	Fruit	Timms et al. (2022)
*Citrus clementina L.*	965	SDG-UC	Fruit	Stanly et al. (2019)
*Citrus limon L.*	263	dUC	Fruit	Raimondo et al. (2015)
*Malus domestica sp.*	181	SDG-UC	Fruit	Trentini et al. (2024)
*Vitis vinifera L.*	55	dUC	Fruit	Pérez-Bermúdez et al. (2017)
*S. lycopersicum L.*	424	SDG-UC	Fruit	Huang et al. (2024)
*S. lycopersicum L.* (piccadilly)	1,752	SDG-UC	Fruit	Bokka et al. (2020)
*Punica granatum L.*	22	SEC	Fruit	Sánchez-López et al. (2022)

Abbreviations: differential ultracentrifugation (dUC); IDG-UC: iodixanol density gradient- ultracentrifugation sucrose density gradient ultracentrifugation; SEC: size exclusion chromatography.

The OGs analyzed provided a robust framework for exploring evolutionary relationships and functional conservation among proteins associated with plant gEVs and PDVs. Notably, we did not identify any OGs common to all the proteomes analyzed, confirming the challenges in defining universal EV markers in plants. However, when focusing specifically on the gEV proteomes, we observed that only the OG8, predominantly composed of Class III peroxidases, was conserved across all species. In contrast, no OGs were found to be consistently shared among the PDV proteomes alone.

**Figure 1:**
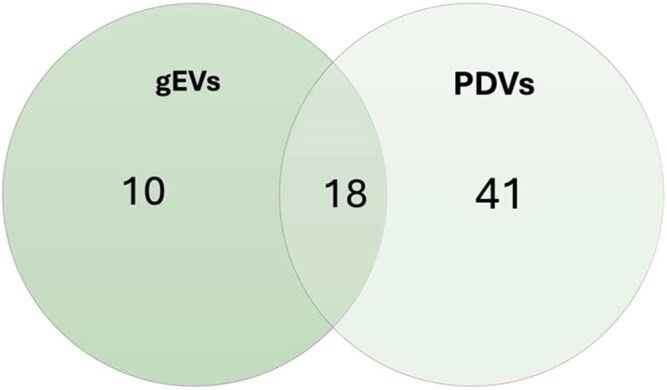
Venn diagram illustrating the distribution of OGs between gEVs and PDVs. A total of 18 OGs are shared between both vesicle types. Ten OGs are most frequently associated with gEVs, while 41 OGs are mainly associated with PDVs.

To refine the dataset and prioritize biologically relevant OGs, we retained for further analysis only those present in at least 66.6% of the EV proteomes within each major category (gEVs and PDVs). This filtering step resulted in a selection of 69 OGs. The distribution of filtered OGs within all proteomes considered is shown in the Table S1. Among these, 10 OGs were predominantly associated with gEV proteomes, 41 were mainly found in PDV proteomes, and 18 were shared between the 2 categories (Fig. 1).

As expected, the PDV category exhibited the highest number of OGs, reflecting the broad diversity of PDV-associated proteins (Table S2). Notably, PDVs displayed a diverse OG profile, with an abundance of proteasome components (OG9, OG10, OG155, OG286), proteases (OG45, OG46, OG76, OG79, OG190), chaperones and Heat Shock Proteins (HSPs) (OG11, OG28, OG218), including Hsp90 family members, small HSPs, and Late Embryogenesis Abundant (LEA) proteins (OG77). PDVs also contained a substantial number of OGs associated with vesicular trafficking and transport regulation, such as Rab GTPases (OG42), clathrin heavy chains (OG84), ADP-ribosylation factors (OG119), and vesicle-associated membrane proteins (VAMPs) (OG124). Additionally, we observed an enrichment of proteins of mitochondrial and plastidial origin, including ATP synthase subunits. Given the highly diverse nature of these PDV-associated proteins, many of which are likely linked to tissue and cell damage, they were not included in further bioinformatic analyses.

**Figure 2:**
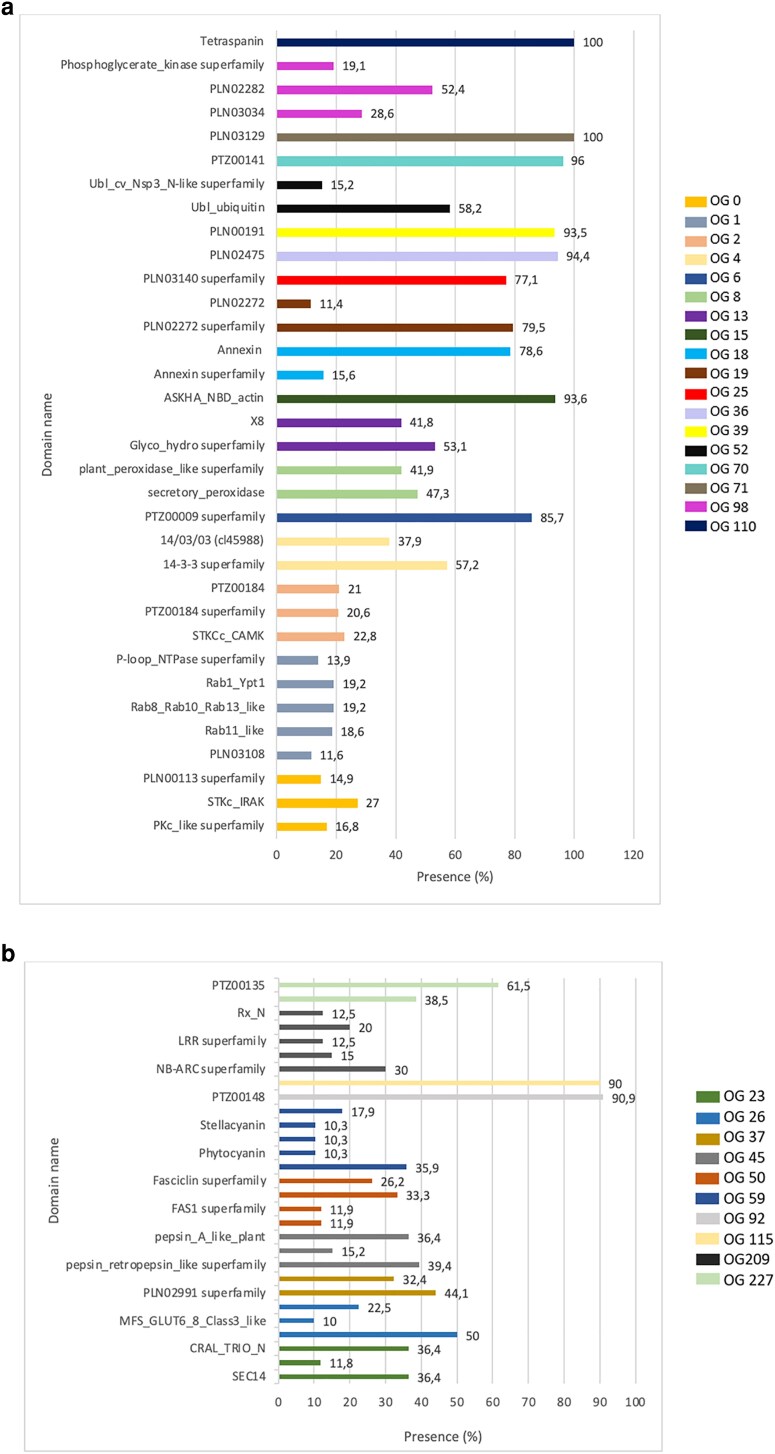
Domain composition of proteins identified in plant extracellular vesicles. (a) Domains commonly shared between genuine EVs (gEVs) and plant-derived vesicles (PDVs). (b) Domains predominantly associated with gEVs. For each OG, the most representative protein domains are displayed. Only domains with a presence exceeding 10% across the OG were included to show the most conserved and functionally relevant features.

The 18 OGs listed in Table 3 and associated domains (Fig. 2a) are shared between gEVs and PDVs, likely representing the most ubiquitous and abundant protein clusters associated with both vesicle types. The distribution of these OGs within the proteomes of the plant species analyzed is shown in Table S3. Notably, this dataset includes large OGs with a high number of proteins and smaller OGs with more specialized functions. Large OGs such as OG0 (349 proteins) include proteins enriched in kinase domains, such as STKc_IRAK and PKc-like. Similarly, OG1 (170 proteins) predominantly contains Rab and P-loop NTPase superfamily proteins, with Ras and Rab11 domains, mainly involved in membrane and vesicles trafficking. OG2 (160 proteins) is enriched in calcium-dependent kinases (CDPKs) and EF-hand domain proteins. The 14-3-3 proteins in OG4 (143 proteins), highly conserved across plant species, regulate signal transduction, the cell cycle, and stress adaptation.

**Table 3:** OGs common in gEVs and PDVs with associated protein counts and predicted functions.

	Protein number	Putative biological function	Representative proteins
OG0	349	Perceiving extracellular signals and activating intracellular responses during growth, development, and immune signaling	Receptor-like protein kinases; Cysteine-rich receptor-like protein kinases 3; Receptor-like serine/threonine-protein kinases
OG1	170	Regulators of membrane trafficking and vesicle-mediated transport in plant cells	P-loop NTPase superfamily, Rab GTPases
OG2	160	Calcium signaling pathways	Calcium-dependent protein kinase; Calmodulin
OG4	143	Signal transduction, cell cycle regulation, and stress responses	14-3-3 proteins
OG6	80	Cellular responses to thermal and other stresses	Heat shock proteins 70
OG8	72	Protection against oxidative damage, peroxidation reactions, and plant adaptation	Peroxidases; L-ascorbate peroxidases
OG13	54	Cleavage of β-1-3 glycosidic bonds in glucans	Glucan endo-1,3-beta-glucosidases
OG15	47	Supporting cytoskeleton structure, cell motility, and cell division	Actins
OG18	44	Calcium regulation, membrane formation, and stress response	Annexin-like proteins
OG19	44	Glucose metabolism and glycolysis	glyceraldehyde-3-phosphate dehydrogenases
OG25	39	Transport of cuticular lipids, phytohormones and secondary metabolites	ATP-binding cassette (ABC) transporters, mostly belonging to G subfamily
OG36	33	Regulation of homocysteine and folate metabolism in plants	5-methyltetrahydropteroyltriglutamate–homocysteine S-methyltransferases
OG39	31	Glucose and carbohydrate metabolism in plants	Enolases
OG52	28	Protein synthesis and post-translational modifications	Ubiquitin-ribosomal proteins; Ubiquitin-NEDD8-like protein
OG70	25	Protein synthesis	Elongation factors 1-alpha
OG71	25	Energy balance and carbon metabolism	Malic enzymes
OG98	21	Cell energy balance and proper functioning of metabolic processes	Phosphoglycerate kinases_ cytosolic
OG110	20	Cell mobility, protein segregation and regulation of cell-extracellular matrix interaction	Tetraspanins

Smaller OGs exhibit more specialized functions, particularly in stress response, structural integrity, and metabolism. OG6 (80 proteins) and OG8 (72 proteins) include proteins from the HSP70 family, as well as peroxidases, with dominant cd00693 and cl00196 peroxidase domains. OG13 (54 proteins) is enriched in glucan endo-1,3-beta-D-glucosidases with the X8 domain (smart00768), facilitating glucan degradation, while OG15 (47 proteins) and OG18 (44 proteins) contain actin (ASKHA_NBD_actin, ASKHA_ATPase-like) and annexin superfamily proteins, with pfam00191 domains. OG19 (44 proteins) consists of glyceraldehyde-3-phosphate dehydrogenase (GAPDH), a key enzyme in glycolysis, characterized by PLN02272 domains with high sequence conservation across species.

Other OGs include proteins involved in transport and metabolic regulation, such as OG25 (39 proteins), OG36 (33 proteins), and OG39 (31 proteins), which contain ABC transporters (PLN03140), methyltransferases (PLN02475), and enolases (PLN00191). OG52 (28 proteins) and OG70 (25 proteins) house ubiquitin and NEDD8-like proteins, with cd01803 (ubiquitin) and cd01806 (NEDD8) domains, as well as elongation factor 1-alpha (EF1A) proteins, indicating strong homology with PTZ00141 domains. OG71 (malic enzymes, PLN03129 domain) and OG98 (3-phosphoglycerate kinases, PLN02282 and PLN03034 domains) further emphasize photosynthetic and metabolic adaptability. Lastly, OG110 (20 proteins) includes tetraspanins (TETs, mostly TET-8, TET-7, and TET-3).

The OGs predominantly associated with gEVs (Table 4) and related domains (Fig. 2b), likely represent core EV components, playing essential roles in vesicle biogenesis, cargo transport, and function.

**Table 4:** OGs prevalently found in gEVs with associated protein counts and predicted functions.

	Protein number	Putative biological function	Representative proteins
OG23	40	Lipid trafficking	Patellins
OG26	39	Transport of sugars, polyols, and other metabolites	Sugar transporters (major facilitator superfamily)
OG37	33	Oxidative processes and copper metabolism	Multicopper oxidases (cupredoxins; L-ascorbate oxidases; laccases); pectinesterases
OG45	30	Protein processing and degradation, Plant defense system	aspartyl protease family proteins
OG50	28	Plant growth, development and structural integrity	fasciclin-like arabinogalactan proteins
OG59	26	Plant growth, defence and communication	phytocyanins (uclacyanin; early nodulin-like protein)
OG92	22	Ribosomal protein (40 s subunit)	Small ribosomal subunit protein eS8
OG115	19	Protein folding, assembly, and repair	Cyclophilins, PPIases
OG209	14	Plant innate immune system	Nucleotide-binding/leucine-rich repeat (NLR) proteins
OG227	13	Ribosomal protein (60 s subunit)	Large ribosomal subunit protein uL10; 60S acidic ribosomal protein P0

The distribution of OGs, related to gEVs, within the proteomes of the plant species analyzed is shown in Table S4. Among these, OG23 (40 proteins) includes proteins primarily involved in lipid transport, membrane trafficking, and signaling. These proteins belong to the SEC14 superfamily, with highly conserved CRAL-TRIO and SEC14 domains, conferring crucial roles in lipid distribution, signaling, and adaptation to environmental stresses, as seen in Patellin-1 (PATL1), Patellin-2 (PATL2), and Patellin-5 (PATL5). OG26 (39 proteins) consists of sugar, polyol, and metabolite transporters, including sugar transport proteins (STP), polyol transporters (PLT), hexose transporters (HT), and major facilitator superfamily (MFS) proteins, all characterized by the MFS_STP domain. Several OGs contain proteins linked to oxidative metabolism, structural integrity, and stress adaptation. OG37 (33 proteins) includes oxidative enzymes such as plastocyanin-like proteins, cupredoxins, laccases, and L-ascorbate oxidases, which share conserved PLN02991 and PLN00044 domains. Similarly, OG45 (30 proteins) consists of aspartic proteases, nepenthesin-like proteins, and peptidase A1 domain-containing proteins, enriched in Pepsin_A_like_plant and Pepsin_Retropepsin_like domains. OG50 (28 proteins) comprises structural proteins such as fasciclin-like arabinogalactan (FLA) proteins, which possess the FAS1 domain, crucial for cell adhesion, structural stability, and cell communication. Functional categories related to plant growth, defence, and environmental communication are found in OG59 (26 proteins), including phytocyanin and early nodulin-like (ENODL) proteins, which OsENODL1-like domains, essential for electron transport and plant-microorganism interactions, as demonstrated in *A. thaliana* (ENODL2, BCB1).

OG92 (22 proteins) and OG115 (19 proteins) are associated with protein synthesis and folding, housing ribosomal proteins (40S, S8, eS8) and peptidyl-prolyl cis-trans isomerases (PPIases), which contain the cyclophilin-like domain (cl00197), playing a key role in protein isomerization and folding. OG209 (14 proteins) includes nucleotide-binding leucine-rich repeat (NLR) proteins, RPP proteins, and AAA+ ATPase domain-containing proteins, central to plant immunity, with domains such as NB-ARC (cl00029) and LRR (cl34836) facilitating pathogen recognition and defence activation. Finally, OG227 (13 proteins) consists of large ribosomal subunit (60S) proteins, including 60S acidic ribosomal protein P0 (uL10), which are part of the Ribosomal_L10_P0 superfamily (cl00376, including PTZ00135) and display high evolutionary conservation across the species considered.

### Prediction of transmembrane domains and signal peptides

**Figure 3:**
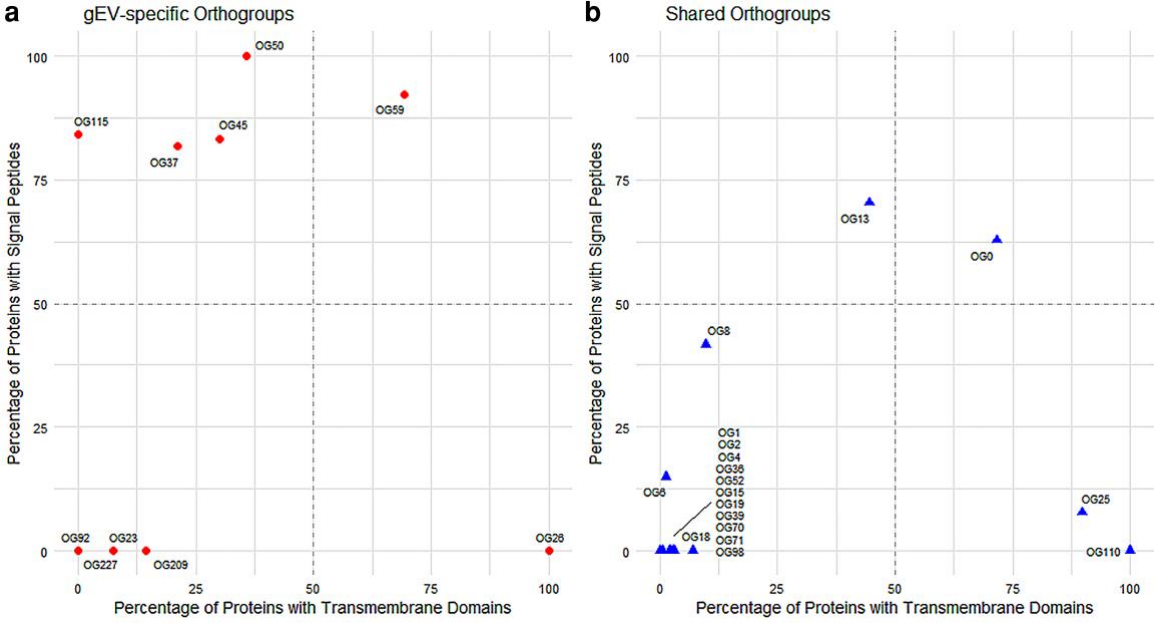
Comparative distribution of signal peptide and transmembrane domain content in OGs associated with plant extracellular vesicles. Scatter plots represent the percentage of proteins containing transmembrane (TM) domains (*x* axis) and signal peptides (SP) (*y* axis) in individual OGs. (a) OGs specifically associated with genuine extracellular vesicles (gEVs). (b) Shared OGs found in both gEV and PDV (plant-derived vesicles) datasets.

To investigate the structural hallmarks of proteins associated with gEVs and those shared with PDVs, we analyzed the presence of transmembrane domains (TM) and signal peptides (SP) across multiple OGs. Data are summarized in Table S5 (gEV-associated OGs) and Table S6 (shared OGs), while a comparative overview of TM and SP is displayed in Fig. 3.

Five OGs uniquely associated with gEVs, such as OG50, OG59, OG45, OG37, and OG115, showed a high proportion of proteins carrying signal peptides (>80%). Functionally, these proteins are involved in plant growth and development (OG50, eg fasciclins), defence and cell communication (OG59, eg phytocyanins), proteolytic activity (OG45, eg aspartyl proteases), oxidative stress and copper metabolism (OG37, eg oxidases), and protein folding (OG115, eg cyclophilins), all roles consistent with vesicle-mediated secretion and extracellular signaling.

In contrast, other gEV-specific OGs, including OG23, OG92, OG209, and OG227, lacked both SP and TM domains entirely. These proteins are primarily associated with lipid trafficking (OG23, eg patellins), protein synthesis (OG92, OG227, eg small and large ribosomal subunits, respectively), and disease resistance (OG209, eg NLR proteins).

Among OGs shared between gEVs and PDVs, OG0 (eg receptor-like protein kinases) and OG13 (eg glucan endo-1_3-beta-glucosidases) presented high TM content (71.6% and 44.4%) coupled with moderate-to-high signal peptide frequency (62.75% and 70.4%), indicating multifunctional roles that include protein phosphorylation via kinases (OG0) and glucan metabolism through enzymatic cleavage of β-glucosidic bonds (OG13). These OGs likely participate in both membrane integration and vesicle-mediated extracellular signaling or enzymatic remodeling. In contrast, OG1 (eg Rab GTPases), OG2 (eg calmodulins), OG4 (14-3-3 proteins), OG19 (GAPDHs), OG36 (5-methyltetrahydropteroyltriglutamate–homocysteine S-methyltransferases), and OG39 (eg enolases) lacked both transmembrane domains and signal peptides, suggesting intracellular or vesicle-lumen localization.

Finally, OG26 and OG25 showed near-complete prevalence of TM domains (100% and 89.7%, respectively) and lacked classical signal peptides. Functionally, these OGs are associated with the transport of sugars and polyols (OG26) and the regulation of intracellular metabolite levels and detoxification processes via ABC transporters (OG25).

### Motif analysis of gEV proteins and those shared with PDVs

A total of 10 motifs were identified in both the full gEV protein dataset and the subset of proteins shared with PDVs. Motif comparison against the PROSITE database using Tomtom (*P*-value < 0.05, highest alignment overlap) allowed us to assign potential functional roles to each motif (Tables 5 and 6). As expected, in both datasets, most motifs were of eukaryotic origin. Several motifs belonging to the gEVs dataset were associated with key cellular functions. For instance, PS00526 (Ribosomal L19E) is involved in ribosome assembly and stabilization, while PS00797 (14-3-3) plays a central role in signal transduction and cell cycle regulation. Motifs such as PS00554 (TEA_1) and PS00350 (MADS_BOX_1) are linked to transcriptional regulation and plant developmental processes, including floral organ identity and root growth. PS01235 (PDXS_SNZ_1), also identified in this dataset, is required for the de novo biosynthesis of vitamin B6, an essential cofactor for a broad range of enzymatic reactions across all domains of life. Notably, PS00540 (FERRITIN_1), a motif involved in iron storage and homeostasis, was uniquely detected in the gEV dataset.

**Table 5:** Conserved motifs identified in gEVs protein dataset. Each motif is characterized by its consensus sequence, corresponding PROSITE ID, sequence pattern, taxonomic distribution, and associated biological function.

Motif number	Motif consensus	Prosite ID	Pattern	Taxonomic range	Function
1	JSWNGIQQRRNSWQDGVYGTNCPIPPGKNFTYILQVKDQIGSFFYFPSLA	PS00526 (RIBOSOMAL_L19E)	Q-[KR]-R-[LIVM]-x-[SA]-x(4)-[CV]-G-x(3)-[IV]-[WK]- [LIVF]-[DNSV]-[PE]	Archaea, Eukaryotes	Ribosome assembly and stabilization of the structure of the RNA
2	FNVEQGKTYRLRVSNVGLQTSLNFRIQNHKMKLVEVEGTHTVQQTYSSLD	PS00994 (FHIPEP)	R-[LIVM]-[GSAT]-E-V-[GSAR]-A-R-F-[STAIV]-L-D-[GSA]-[LM]-P-G-K-Q-M-[GSA]- I-D-[GSA]-[DAE]	Prokaryotes (Bacteria)	Bacterial motility, secretion systems, pathogenesis and host interactions
3	LRQATKQALQLLQDNYPEFVAKQVFINVPWWYLAFYKMISPFLTQRTKSK	PS00797 (1433_2)	Y-K-[DE]-[SG]-T-L-I-[IML]-Q-L-[LF]-[RHC]-D-N-[LF]-T-[LS]-W-[TANS]-[SAD]	Eukaryotes	Signal transduction and cellular regulation
4	DPHIEEQFGSGRLLACISSRPGQCGRADGYILEGKELEFYMKKLQKKKGK	PS00540 (FERRITIN_1)	E-x-[KR]-E-x(2)-E-[KR]-[LF]-[LIVMA]-x(2)-Q-N-x-R-x-G-R	Eukaryotes	Iron storage and homeostasis
5	YNLRDAVARSTVQVYPKSWTAIYIALDNVGMWNLRSEFWARQYLGQQLYL	PS00994 (FHIPEP)	R-[LIVM]-[GSAT]-E-V-[GSAR]-A-R-F-[STAIV]-L-D-[GSA]-[LM]-P-G-K-Q-M-[GSA]- I-D-[GSA]-[DAE]	Prokaryotes (Bacteria)	Bacterial motility, secretion systems, pathogenesis and host interactions
6	LKDERSDVILLKFLRARDFKVKEAFAMLKNTVQWRKEFGID	PS00812 (GLYCOSYL_HYDROL_F8)	A-[ST]-D-[AG]-D-x(2)-[IM]-A-x-[SA]-[LIVM]-[LIVMG]-x-A-x(3)-[FW]	Prokaryotes (Bacteria)	Contributes to cleaving glycosidic bonds in polysaccharides
7	GDIYPLGVPQQGILINGQFPGPDIN SVTNBNLIINVFNSLD	PS00522 (DNA_POLYMERASE_X)	G-[SG]-[LFY]-x-R-[GE]-x(3)-[SGCL]-x-D-[LIVM]-D-[LIVMFY](3)-x(2)-[SAP]	Eukaryotes, Prokaryotes (Bacteria), Eukaryotic viruses	DNA synthesis and repair
8	RILDVVYNASNNELVRTQTLVKSAIVQVDAAPFKQWYLQHY	PS00554 (TEA_1)	G-R-N-E-L-I-x(2)-[YH]-I-x(3)-[TC]-x(3)-R-T-[RK](2)-Q-[LIVM]-S-S-H-[LIVM]- Q-V	Eukaryotes	Regulation of gene expression by binding specific DNA sequences, particularly involved in developmental and cellular processes
9	WSLNQARSIRTNLTASGPRPNPQGSYHYG	PS01235 (PDXS_SNZ_1)	[LV]-P-[VI]-[VTPI]-[NQLHT]-[FL]-[ATVS]-[AS]-G-G-[LIV]-[AT]-T-P-[AQS]-D- [AGVS]-[AS]-[LM]	Archaea, Eukaryotes, Prokaryotes (Bacteria)	De novo synthesis of vitamin B6, essential cofactor in numerous enzymatic reactions related to amino acid metabolism, stress responses and plant development
10	MGISRDSMHKRRATGGKKKAWRKKRKYELGRQPANTKLSSN	PS00350 (MADS_BOX_1)	[RGS]-x-[RKS]-x(5)-[IL]-x-[DNGSK]-x(3)-[KR]-x(2)-T-[FY]-x-[RK](3)-x(2)-[LIVM]-x-[KE]-K-[AT]-x-[EQ]-[LIVM]-[STA]-x-L-x(4)-[LIVM]-x-[LIVM]- [LIVMT]-[LIVM]-x(6)-[LIVMFC]-x(2)-[FYLS]	Eukaryotes	Crucial roles in plant development, especially in flowering, fruit development, and organ identity

**Table 6:** Conserved motifs identified in proteins shared between gEVs and PDVs. Each motif is characterized by its consensus sequence, corresponding PROSITE ID, sequence pattern, taxonomic distribution, and associated biological function.

Motif number	Motif consensus	Prosite iD	Pattern	Taxonomic range	Function
1	YEILNSPDRACNLAKQAFDEAIAELDTLGEESYKDSTLIMQLLRDNLTLW	PS00755 (SECY_1)	[GSTL]-[LIVMFK]-[LIVMFCA]-x-[LIVMF]-[GSAN]-[LIVM]-x-P[LIVMFYN]-[LIVMFY]- x-[AS]-[GSTQD]-[LIVMFAT](3) -[EQ]-[LIVMFA](2)	Archaea, Eukaryotes, Prokaryotes (Bacteria)	Translocation of proteins across or into membranes
2	IIHRDIKSSNILLDEDFNAKVADFGLAKL	PS00220 (ANION_EXCHANGER_2)	[FI]-L-I-S-L-I-F-I-Y-E-T-F-x-K-L	Eukaryotes	Transport of anions across membranes
3	GTFGYLAPEYAMTGQLTEKSDVYSFGVVL	PS01235 (PDXS_SNZ_1)	[LV]-P-[VI]-[VTPI]-[NQLHT]-[FL]-[ATVS]-[AS]-G-G-[LIV]-[AT]-T-P-[AQS]-D- [AGVS]-[AS]-[LM]	Archaea, Eukaryotes, Prokaryotes (Bacteria)	De novo synthesis of vitamin B6, essential cofactor in numerous enzymatic reactions related to amino acid metabolism, stress responses and plant development
4	LDWKQRLKIAJGAARGLEYLH	PS01327 (MSCL)	[KR]-G-N-[LIVM](2)-D-[LIVM]-A-[LIVM]-[GA]-[LIVM](3)-G	Prokaryotes (Bacteria)	Regulation of osmotic pressure changes within the cell
5	KLQIWDTAGQERFRTITSAYY RGAVGALLVYDVTDRESFNN	PS00547 (TRANSGLUTAMINASES)	[GT]-Q-[CA]-W-V-x-[SA]-[GAS]-[IVT]-x(2)-T-x-[LMSC]-R-[CSAG]-[LV]-G	Eukaryotes	Contribution to protein stabilization and tissue structure
6	LTVEERNLLSVAYKNVIGARRAS WRIJSSIEQKEESRGNEDHVKLIKEYR	PS00554 (TEA_1)	G-R-N-E-L-I-x(2)-[YH]-I-x(3)-[TC]-x(3)-R-T-[RK](2)-Q-[LIVM]-S-S-H-[LIVM]- Q-V	Eukaryotes	Regulation of gene expression by binding specific DNA sequences, particularly involved in developmental and cellular processes
7	FYLKMKGDYHRYLAEFKTGDER KEAAESTLKAYKAAQDIANAELAPTHPI	PS00994 (FHIPEP)	R-[LIVM]-[GSAT]-E-V-[GSAR]-A-R-F-[STAIV]-L-D-[GSA]-[LM]-P-G-K-Q-M-[GSA]- I-D-[GSA]-[DAE]	Prokaryotes (Bacteria)	Bacterial motility, secretion systems, pathogenesis and host interactions
8	HRNLVRLLGYCVEGDERJLVY	PS01205 (T4_DEIODINASE)	R-P-L-[IV]-x-[NS]-F-G-S-[CA]-[TS]-[CU]-P-x-F	Eukaryotes	Regulation of metabolism and development
9	YDYLFKLVLIGDSGVGKSCLLLR FTDNSFLLSSISTIGVEF	PS01290 (ER)	Y-D-I-[SA]-x-L-[FY]-x-F-[IV]-D-x(3)-D-[LIV]-S	Eukaryotes	Putative role in the cell cycle
10	REZNVYMAKLAEQAERYEE MVEFMEKVAK	PS01167 (RIBOSOMAL_L17)	[IL]-x-[STV]-[GT]-x(2)-[KR]-x-[KRAF]-x(6)-[DE]-x-[LIMV]-[LIVMT]-[TE]-x- [STAG]-[KR]	Eukaryotes, Prokaryotes (Bacteria)	Structural component of the large ribosomal subunit, essential for protein synthesis.

Additionally, 2 motifs of clear prokaryotic origin were also detected. PS00994 (FHIPEP) is typically linked to bacterial motility and secretion systems, mediating the translocation of specific proteins across bacterial membranes through a mechanism that does not require signal peptide cleavage. PS00812 (GLYCOSYL_HYDROL_F8), on the other hand, is involved in the degradation of polysaccharides.

In the subset of proteins shared between gEVs and PDVs, 10 motifs were also identified. Three of these overlapped with motifs from the gEV dataset: PS01235 (PDXS_SNZ_1), PS00554 (TEA_1), and again PS00994 (FHIPEP), whose recurrence in both datasets points to a conserved functional role, potentially related to vesicle-mediated transport.

The other shared motifs were associated with additional functional signatures. PS00755 (SECY_1) is involved in protein translocation across membranes; PS00547 (TRANSGLUTAMINASES) catalyzes calcium-dependent protein cross-linking; and PS01205 (T4_DEIODINASE) participates in hormone metabolism. PS01290 (ER) is potentially related to cell cycle control while PS01167 (RIBOSOMAL_L17) represents a structural ribosomal component essential for protein synthesis. PS00220 (ANION_EXCHANGER_2) contributes to anion transport and PS01327 (MSCL), a mechanosensitive channel domain of prokaryotic origin, is likely involved in the regulation of osmotic pressure.

## Discussion

### Challenges in isolating and defining plant EVs

In recent years, plant EVs have attracted significant attention in plant biology mainly due to their crucial roles in defence, cell wall remodeling, and plant–microbe interactions (Zhou et al. 2022). However, a deep understanding of their biological function remains challenging, especially for the difficulties to collect pure EV preparations from plant tissues and organs.

Advances in purification techniques, size-exclusion chromatography (SEC) and tangential-flow filtration (TFF) have improved the separation and reproducibility of vesicle subpopulations compared with conventional differential ultracentrifugation (Lian et al. 2022; Huang et al. 2025). The choice of the starting material is equally critical. Indeed, EVs obtained from extracellular fluids, such as apoplastic wash fluids or conditioned media of cell and tissue cultures, are generally enriched in gEVs. In contrast, vesicles isolated from tissue macerates, here referred to as PDVs, represent a heterogeneous population that may include intracellular vesicles, membrane fragments, and other lipidic structures.

Although both gEVs and PDVs can be valuable for research and applications, they serve different purposes. gEVs are particularly suited for studying mechanisms of intercellular communication, stress responses, and signaling, as they reflect the physiological release of vesicles occurring under both normal and stress-induced conditions. PDVs, on the other hand, represent a heterogeneous vesicle population that, although less reflective of physiological secretion processes, holds strong biotechnological potential. Their abundance makes them attractive as scalable sources of vesicle-like nanoparticles for applications in drug delivery, cosmetics, and agriculture.

Nevertheless, contamination by protein complexes and cytosolic components remains a major limitation. The establishment of standardized isolation protocols and robust biochemical and biophysical characterization pipelines is therefore essential to distinguish genuine gEVs and PDVs from co-purified contaminants and to ensure reproducibility across studies. Continued methodological refinement will ultimately enable a more accurate definition of plant EV subtypes and their biological and applicative relevance.

### Comparative proteomics reveals distinct cargo profiles in gEVs and PDVs

Proteomic analyses are instrumental in uncovering the EV biocargo. Such approaches are still scant in plant EV field, with some interesting reports conducted in a limited number of species (Pinedo et al. 2021; Rodríguez de Lope et al. 2025). However, datasets of plant proteins associated with vesicles isolated from extracellular fluids like apoplast, plant in vitro cultures, and disrupted tissues such as fruit juices are rapidly and independently accumulating. Beyond identifying the repertoire of cargo proteins in plant species, comparative proteomics of gEVs and PDVs facilitates conserved protein discovery and helps to elucidate the mechanisms of EV biogenesis, including loading processes and secretion, and biological roles.

To the best of our knowledge, this analysis is the first that distinctly considers and compares 22 proteome datasets from genuine EVs and PDVs. Our goal was to identify and systematically characterize the functions associated with each vesicle type, examining molecular features such as protein domains, signal peptides, membrane localization, and conserved motifs, to uncover distinct proteomic signatures of gEVs and PDVs. In parallel, we also aimed to identify potential contaminants based on low conservation and high variability across datasets within our comparative framework.

Orthogroup distribution across the analyzed proteomes showed considerable variability, with no single OG conserved across all datasets, reflecting the difficulty in defining universal protein markers for plant EVs. Even within gEV proteomes, considered more representative of naturally secreted vesicles, only OG8, predominantly composed of Class III peroxidases, was consistently conserved. The absence of universally shared OGs across both gEV and PDV proteomes is likely the result of several factors, including the vast genetic diversity among plant species, organ- and tissue-specific molecular differences, and technical and methodological variations in EV isolation and protein extraction protocols. Moreover, differences in proteome quality may further contribute to these discrepancies. Altogether, these observations point to the need for greater standardization and more systematic comparisons to enable the identification of reliable, plant-specific EV markers.

More interestingly, our analysis revealed that certain protein clusters and domains are consistently detected across various plant species in both gEVs and PDVs, while others are predominantly found in either gEVs or PDVs. Regarding the proteins mostly found in PDVs, we observed the broad representation of proteasome components (eg proteasome subunit alpha, 26S Proteasome), proteases (cysteine proteases), small chaperones (eg Hsp20/SHSP domain-containing proteins), and some HSPs (mainly HSP90), likely reflecting their activation and release following tissue damage. Moreover, we also found the presence of VAMPs such as VAMP711, VAMP713, VAMP714 known to be central for the intracellular trafficking, particularly in mediating vesicle fusion events between the Golgi apparatus, tonoplast, and the plasma membrane (Uemura et al. 2004; Fujiwara et al. 2014; Xue et al. 2018; Gu et al. 2021).

The heterogeneity observed in PDV-associated proteins suggests that their presence may be, in part, a consequence of cellular disruption rather than actively packaging linked to specific functional roles in plant EVs. However, the possibility of species-specific or tissue/organ-dependent roles for the reported protein clusters identified mostly in PDVs cannot be ruled out and may be further elucidated through future investigations.

Another key observation is that OG size varies significantly between those exclusive to genuine EVs and those shared with PDVs. Larger OGs containing >100 proteins (eg OG0, OG1, OG2, OG4) are predominantly shared among gEVs and PDVs and likely represent conserved and highly expressed proteins with fundamental cellular roles. For instance, examining the largest OGs, it is worth mentioning that members of OG0, including diverse receptor-like kinases (RLKs) and serine/threonine protein kinases, play key roles in plant immunity, development, hormone signaling, and stress adaptation (Kileeg and Mott 2025). The prevalence of these kinases in both gEVs and PDVs may reflect their high cellular abundance and widespread involvement in signaling cascades. However, their recurrent also points toward a potential functional significance in the EV context. Rather than being incidental cargo, their consistent presence suggests a regulated inclusion in vesicles, possibly contributing to signal relay between cells. In animal systems, the EV-mediated transfer of kinases has been shown to influence recipient cell behavior, modulating signaling networks and determining cell fate (Irmer et al. 2023). However, such EV-mediated signaling mechanisms remain largely unexplored in plants so far.

Similarly, the dominance of Rab GTPases in the second-largest OG reinforces the view that plant EVs may rely on conserved trafficking machinery. Rab proteins are central to vesicle formation, targeting, and fusion and their occurrence across multiple Rab families (eg, RABA2a, RABD2a, RABG3b, Rab11) supports the hypothesis that EV biogenesis in plants involves tightly regulated processes (Homma et al. 2021; Tripathy et al. 2021). According to Vesiclepedia, 40 Rab isoforms have been characterized in EVs derived from various mammalian sources, including *Homo sapiens*, *Mus musculus*, and *Rattus norvegicus*. While the role of all Rab members in EV biology remains under investigation, some, such as Rab11, which was also identified in our analysis, play a crucial role in EV biogenesis interacting with ESCRT-III proteins, thus facilitating EV sorting and release (Marie et al. 2023). Comparative sequence analysis further supports the evolutionary conservation of this pathway, showing 71.56% amino acid identity between human *Rab11A* and its *A. thaliana* ortholog *RABA2a (Rab11C),* suggesting that Rab11-like GTPases may perform analogous roles in plant EV trafficking and secretion. Further investigations are needed to prove whether this Rab11 play comparable function in plant EV biogenesis.

Other key functions are included in the OG2 and OG4 with representative members of calcium signaling pathway and 14-3-3 clusters, respectively. Calcium signaling is a ubiquitous second messenger pathway in plant cells, mediating a variety of physiological responses including vesicle fusion, stress response, and developmental processes (Tuteja and Mahajan 2007). More in detail, several proteins in OG2 show homology to different classes of proteins including calmodulins, calcineurin B-like proteins (CBLs), and calcium-dependent protein kinases (CDPKs). Their presence in EVs suggests a possible role in modulating calcium-dependent signaling pathways in recipient cells or tissues, but also a possible role in immunity (Yang et al. 2023). In mammalian systems, EV-associated calcium-binding proteins have been reported as well and recently associated with EV cargo sorting (Ono et al. 2023).

OG4, composed predominantly of 14-3-3 proteins, is also noteworthy due to the central regulatory role these proteins play across eukaryotes. The 14-3-3 s function as adaptors or scaffolds in signaling pathways, interacting with phosphorylated client proteins to regulate a wide spectrum of processes, including metabolism, hormone perception (eg ABA, GA), protein trafficking, and stress signaling (Dovrat et al. 2014; Kaplan et al. 2017). Within the context of EVs, 14-3-3 proteins may serve structural roles, facilitate cargo sorting, or act as signaling mediators in recipient cells (Fraser et al. 2013). Given their strong evolutionary conservation, it is likely that they fulfill conserved functions in plant EV biology.

Altogether, the recurrent enrichment of conserved signaling modules points to a deeper layer of functional specialization within plant EVs, raising the exciting possibility that these vesicles serve as mobile signaling vehicles between the cells.

Beyond the complex OGs, it is worth noting that several small OGs (comprising fewer than 50 proteins) are shared between EVs and PDVs. For instance, we found proteins such as GAPDH and enolase involved in glucose and carbohydrate metabolism in plants (OG19, OG39), proteins involved in supporting cytoskeleton structure (eg actin), proteins involved in the process of protein synthesis (eg Elongation factors 1a), and important membrane proteins such as annexins and different members of Tetraspanin (TET) family proteins, such as TET7, TET8, and TET3-like, which are known to play critical roles in many membrane associated processes (Zaffagnini et al. 2013; Wang et al. 2015; Jakobsson et al. 2018; Zheng et al. 2025). Overall, these proteins have been largely described in mammal exosomes as well. In particular, tetraspanins such as CD9, CD37, CD63, CD81, and CD82 are found on the membranes of EVs and widely recognized as EV marker proteins (Kowal et al. 2016; Jeppesen et al. 2019). Likewise, the EV-associated proteins TET8 have been identified and employed as candidate markers for EVs, especially in *A. thaliana* (Cai et al. 2018). Importantly, TET8 has been directly implicated in exosome secretion in *A. thaliana*, highlighting its pivotal role in plant EV biology (Liu et al. 2024).

### A focus on the core protein clusters associated with *bona fide* plant EVs

The OGs, predominantly enriched in gEVs, are likely to represent core components of the plant EV physiological functions. Importantly, these protein clusters have been recurrently identified across a wide range of plant species and through independent EV isolation methods, including apoplastic fluid extraction, hairy root culture media, and plant cell suspension cultures. This cross-species and cross-source reproducibility reflects a conserved and biologically meaningful extracellular role of this protein biocargo. Many of these EV-enriched proteins belong to small OGs, such as OG23, OG26, OG37, and OG45, further suggesting selective packaging rather than passive release, pointing to functional specialization and evolutionary conservation of plant EV-mediated communication.

While the proteins shared between gEVs and PDVs tend to reflect more general and constitutive functions, the conserved protein repertoire of gEVs may provide specific information to EV-related processes.

For instance, the presence of SEC14-domain–containing proteins such as the Patellin family (PATL1, PATL2, PATL5) supports the idea that EV biogenesis in plants involves dedicated lipid trafficking and membrane remodeling machinery, analogous to annexins and flotillins in mammalian systems (Dixson et al. 2023).

Similarly, the detection of sugar and metabolite transporters, including STPs and members of the MFS family, points toward a role for EVs in mediating solute exchange between cells or compartments. Though traditionally unlinked to EVs, their consistent detection across samples hints at a potential role in nutrient signaling, symplastic-to-apoplastic metabolite exchange, or even microbiome modulation via EVs. These transporters may enable EVs to act as vectors for solute redistribution under stress or during intercellular coordination, echoing similar nutrient-related cargo seen in mammalian vesicles. For instance, in mammals, exosomes released under oxidative, ischemic, and metabolic stress have been shown to carry solute transporters that mediate metabolic adaptation and promote cell survival (Hirpara et al. 2024; Czpakowska et al. 2025). Proteins involved in oxidative metabolism and ROS detoxification are well-represented in OG37 (33 proteins), including monocopper oxidases, cupredoxins, and laccases. These enzymes play central roles in lignin biosynthesis, phenolic metabolism, and redox signaling, suggesting that their export via EVs may contribute to cell wall remodeling, oxidative stress adaptation, or defence priming in the extracellular matrix.

OG50 (28 proteins) comprises FLA proteins, characterized by FAS1 domains involved in cell adhesion, cell wall architecture, and intercellular signaling. These glycoproteins, abundant in the plant extracellular space, may serve as anchoring or targeting components within EVs, helping vesicles interface with the cell wall or recipient cells. FAS1 domains are evolutionarily ancient and found across kingdoms, paralleling the adhesive and targeting roles of integrins and tetraspanins in mammalian EVs.

Perhaps most compelling is the presence of OG59 associated with immune surveillance and host–microbe communication, including early nodulin-like (ENODL) proteins and phytocyanins, originally studied in symbiotic contexts but now increasingly recognized for their roles in broader plant–microbe interactions (Denancé et al. 2014). Their EV association raises the possibility that plants deploy EVs as immunological messengers, capable of both perceiving and responding to microbial cues in the extracellular space (Rutter and Innes 2017; De Palma et al. 2020). This is further reinforced by the detection of NLR immune receptors and AAA+ ATPases, components of the canonical immune machinery, as well as many proteases (aspartic proteases, nepenthesin-like enzymes, and proteins with Pepsin_A_like and Retropepsin-like domains), which may participate in the extracellular remodeling of proteomes during infection or damage.

Finally, drawing a parallel with mammalian EVs, we identified 2 OGs, OG92 and OG227, enriched in ribosomal proteins, and OG115, comprising peptidyl-prolyl isomerases (cyclophilins, PPIases), such as cyclophilins. Although ribosomal proteins were historically dismissed as contaminants in EV preparations, accumulating evidence now points to their functional role in extracellular communication (Ochkasova et al. 2023; Graifer et al., 2025). Similarly, PPIases, traditionally known for their role in protein folding (Dunyak and Gestwicki 2016), may exert these functions in EVs by stabilizing protein cargo, assisting in extracellular protein folding, or participating in stress-responsive signaling pathways. Their repeated identification across species suggests a conserved, functionally relevant role in the plant EV molecular toolkit.

### Decoding EV proteome cargo: loading mechanisms and secretion pathways

EV cargo loading is a complex and still poorly understood mechanism in plants. However, evidence from mammalian systems provides useful parallels, as selective cargo incorporation in animal EVs is mediated by both ESCRT-dependent and ESCRT-independent pathways regulated by tetraspanins, ceramide-enriched lipid microdomains, and Rab GTPases, together with RNA-binding proteins that direct the sorting of specific miRNAs (Colombo et al. 2014; Mathieu et al. 2019). The presence of homologous components in plant EVs, including ESCRT complexes, tetraspanins (TET8/TET9), and SNARE proteins (PEN1, VAMP721/722), suggests that analogous principles of cargo selection and vesicle formation may be evolutionarily conserved across kingdoms (Théry et al. 2018; Ambrosone et al. 2023). Other clues into how proteins are selectively incorporated into plant EVs can be drawn from their structural characteristics, particularly the presence or absence of transmembrane (TM) domains and signal peptides, which often reflect their intracellular localization, trafficking routes, and ultimate positioning within the vesicle. Proteins containing TM domains are likely embedded in the lipid bilayer of the EV membrane and may serve not only as cargo but also as active participants in vesicle formation, targeting, or signaling. Interestingly, despite their expected abundance in membrane-derived vesicles, relatively few OGs contain proteins with TM domains, possibly reflecting the technical challenges in solubilizing and detecting less abundant or highly hydrophobic transmembrane proteins in standard proteomic workflows. Such proteins include sugar transporters, ABC transporters, receptor-like protein kinases, and membrane-anchored enzymes (eg Glucan endo-1,3-β-D-glucosidases such as Q56WE6_ARATH, Q9M2T6_ARATH). Their stable association with the EV membrane makes them attractive candidates for vesicle tracking and as molecular scaffolds for bioengineering approaches aimed at enhancing EV targeting specificity or functionalization.

In contrast, proteins lacking TM domains are likely sequestered within the vesicle lumen, representing a protected reservoir of biologically active molecules. These luminal cargos are of particular interest for both fundamental plant biology and applied biotechnology, as they may be directly delivered to recipient cells, where they can influence physiological responses. For example, based on our observations, small ribosomal subunit proteins, large ribosomal subunit proteins, P-loop NTPase superfamily, Rab GTPases, calmodulins, and enolases are likely to reside within the EV lumen.

Remarkably, the loading of proteins into EVs is closely tied to their cellular secretion pathways. Traditionally, proteins possessing signal peptides (SPs) are directed into the endoplasmic reticulum (ER) and secreted via the classical ER–Golgi-dependent Sec/SPI pathway. However, numerous proteins lack SPs and are secreted through nonclassical or unconventional secretion (UPS) mechanisms that bypass the ER–Golgi system (Ding et al. 2014). These proteins are often released under stress or developmental cues and are functionally active in the extracellular space, particularly in plant defense and signaling contexts (Cohen et al. 2020).

In our study, we found that many OGs in gEVs and PDVs, such as OG1 (eg Rab GTPases), OG2 (eg calmodulins), OG4 (eg 14-3-3 proteins), OG23 (eg patellins), OG15 (eg actins), OG18 (annexins), O19 (glyceraldehyde-3-phosphate dehydrogenases), OG26 (major facilitator superfamily) and others, lack conserved signal peptides, supporting the notion that EVs facilitate unconventional secretion of leaderless proteins. These OGs include proteins involved in lipid trafficking, translation, and immune response, highlighting EVs as a key route for exporting cytosolic proteins that are otherwise not secreted by canonical pathways.

Conversely, several gEV-associated protein clusters such as OG50 (eg fasciclins, FAS1), OG59 (eg phytocyanins and early nodulin-like proteins), OG45 (eg aspartyl proteases), OG37 (copper oxidases, cupredoxins and L-ascorbate oxidases), and OG115 (PPIases/cyclophilins) do contain SPs, raising questions about the specificity of their association with EVs. It is possible that such proteins are secreted independently and only later adhere to the vesicle surface or co-purify during EV isolation. However, their consistent detection in EV fractions across many plant species suggests that EVs may also actively recruit proteins from the classical secretion pathway, potentially to enhance their extracellular stability, targeting, or bioactivity. These observations imply that EVs are not limited to mediating unconventional secretion but may also play an active role in redistributing conventionally secreted proteins. If experimentally validated, this dual capacity would reinforce the concept of EVs as dynamic platforms for fine-tuning extracellular signaling, stress adaptation, and intercellular communication.

### Conserved functional signatures of plant EVs

Finally, our analysis also identified conserved motifs in plant EV proteomes, unveiling molecular features underlying vesicle biogenesis, cargo sorting, and intercellular communication. These motifs cover a broad spectrum of biological roles, many of which are directly or indirectly associated with transport processes, structural integrity, and signaling, key aspects of EV function. Of particular interest are motifs typically associated with transcriptional regulation such as TEA_1 (PS00554) and MADS_BOX_1 (PS00350), which were detected in proteins predominantly found in gEVs. While the functional activity of such transcription factors in extracellular contexts remains to be experimentally verified, their presence points to a potential mechanism for long-distance signaling in developmental or stress-response programs, a concept that, although well-documented in mammalian systems remains to be fully explored in plant EVs (Zhou et al. 2018; Dissanayake et al. 2024).

The PS01235 (PDXS_SNZ_1) motif suggests a conserved role for EVs in transporting metabolic enzymes or cofactors implicated in redox balance, stress responses, and cellular homeostasis (Lo et al. 2024). The gEV-specific proteome also revealed the presence of PS00540 (FERRITIN_1), a conserved motif associated with storage and homeostasis. This finding raises the possibility that plant EVs may participate in iron handling or redox regulation, potentially contributing to cellular defence mechanisms against iron-induced oxidative stress or mediating iron mobilization in the extracellular environment. While ferritin secretion via extracellular vesicles has been reported in mammalian systems (Truman-Rosentsvit et al. 2018) its occurrence in plant-derived EVs has yet to be functionally characterized, suggesting an intriguing avenue for future research.

The detection of FHIPEP (PS00994), a motif typically linked to bacterial secretion and motility, and PS00812 (GLYCOSYL_HYDROL_F8), belonging to glycosyl hydrolase family 8 also raises intriguing possibilities. These prokaryotic-associated motifs may reflect functional convergence, horizontal gene transfer, or alternatively co-isolation of microbial vesicles or endophytic/epiphytic microbiota (Rutter and Innes 2017; Regente et al. 2017). Their biological relevance in the plant EV context remains to be clarified, further underscoring the need for rigorous purification and characterization (Théry et al. 2018).

Within the subset of shared proteins between gEVs and PDVs, motif analysis confirmed the recurrence of 3 motifs (PDXS_SNZ_1, TEA_1, and FHIPEP), strengthening the hypothesis of a conserved vesicle-associated core proteome. Additional shared motifs, including PS00755 (SECY_1), a sequence conserved across all domains of life and essential for translocating proteins across membranes, and PS00220 (ANION_EXCHANGER_2), suggest a contribution of vesicle protein loading, solute transport, and environmental sensing, based on their known roles in other biological systems. The identification of MSCL (PS01327), a mechanosensitive channel domain of bacterial origin, also raises questions about the presence of horizontal gene transfer events or microbial protein carryover during vesicle isolation.

Taken together, this comparative motif analysis reveals an overlooked but functionally rich signature within plant EVs, one that not only reflects conserved biological processes but also offers evolutionary parallels to mammalian systems, and even possible links to microbial biology.

## Conclusion

Our study delivers the most extensive systematic and comparative analysis of published plant EV proteomes, sorted by their true extracellular origin and considering the potential contribution of other types of vesicles such as PDVs. First, this work was essential to integrate and rationalize the currently fragmented proteomic data on plant EVs, enabling the identification of conserved molecular features that individual datasets could not reveal. Then, through this integrative approach, we delineate a conserved set of proteins shared across different EV types (eg. receptor-like kinases, Rab GTPases, calcium-dependent protein kinases, calmodulins, 14-3-3 proteins, HSP 70, peroxidases, glucan endo-1,3-β-glucosidases, actins, annexin-like proteins, ABC transporters, GAPDH, phosphoglycerate kinases, enolases, elongation factors 1-alpha, tetraspanins, and others) and identify proteins prevalently associated with gEVs, such as patellins, sugar transporters, multicopper oxidases, pectinesterases, aspartyl proteases, FLA proteins, phytocyanins, and NLR proteins. These results provide a solid foundation for guiding future functional studies aimed at validating the biological significance of the most promising protein markers and conserved motifs. Follow-up experimental approaches, such as CRISPR/Cas-mediated knockouts, live-cell imaging, or protein tracking assays, will be instrumental to confirm their mechanistic roles in EV plant biology.

Interestingly, both gEV- and PDV-associated protein clusters display clear translational potential. For instance, VAMPs (eg Rab GTPases, tetraspanins, and receptor-like kinases) could be exploited for EV engineering by fusion with proteins that enhance traceability (eg green fluorescent proteins), strengthen plant defence responses (eg pathogen-recognition receptors or antimicrobial peptides), or enable targeted delivery to specific cell types for therapeutic purposes. Likewise, stress-related chaperones, proteasome subunits, 14-3-3- and calcium-signaling proteins provide molecular entry points to explore EV functions in mitigating environmental stress. Not least, our analysis confirmed that EVs carry proteins involved in plant defence and immunity. This molecular arsenal could be further activated, for example, through elicitation by using plant biotechnological platforms, to harness EVs as innovative tools for sustainable crop protection.

## Materials and methods

### Study selection and proteomic data processing

An extensive database search was performed across PubMed, Scopus, and Google Scholar up to June 2024. Studies were considered eligible if they investigated EVs obtained by either (i) direct collection from the apoplast or cell/tissue culture medium (genuine extracellular vesicles, gEVs) or (ii) tissue disruption (plant-derived vesicles, PDVs). We also included the proteome of EVs isolated by SEC (Gen 2 qEV columns, IZON Science) from conditioned media of cardoon (*Cynara cardunculus*) suspension cell cultures. We deposited this proteome to the ProteomeXchange Consortium via the PRIDE partner repository (Perez-Riverol et al. 2022) with the dataset identifier PXD059086.

To ensure a balanced comparison, a similar number of studies were included for each isolation method. In total, 22 proteomic datasets were selected: 10 from gEVs and 12 from PDVs. To broaden evolutionary comparisons, plant species from different botanical families were included. The total number of proteins identified in each dataset is reported in Tables 1 and 2, together with the corresponding plant species, EV isolation methods, tissue sources, and references. To reduce dataset size variability, comparative analyses were performed at the OG level, which normalizes protein representation by grouping evolutionarily related sequences across species and allows functional comparisons independent of absolute protein counts. Following study selection, proteomic data were systematically retrieved and processed. Specifically, protein accession numbers from each dataset were collected and annotated using the UniProt Knowledgebase (https://www.uniprot.org/id-mapping), providing information on sequences, structures, and functions. Corresponding protein sequences were then downloaded in FASTA format and used for downstream analyses.

### Orthology inference analysis

Orthology inference was performed using OrthoFinder version 2.4.0 (Emms and Kelly 2019). OrthoFinder was used to carry out sequence alignment and identify OG across the analyzed plant proteomes. OGs were defined according to OrthoFinder's default criteria, where each OG represents a set of orthologous proteins descended from a single ancestral gene in the last common ancestor of the analyzed species. The retrieved protein sequences for all species were provided as input, and OrthoFinder was used to identify single-copy OGs. Subsequently, based on protein names, we quantified the number of orthologs identified in the synteny analysis that were assigned to each OrthoFinder orthogroup.

### Conserved domain analysis

To investigate conserved domains across different protein families, the Conserved Domain Database (CDD; Marchler-Bauer et al. 2015) maintained by the National Center for Biotechnology Information (NCBI) was used. Specifically, the Batch CD-Search tool (https://www.ncbi.nlm.nih.gov/Structure/bwrpsb/bwrpsb.cgi) was employed using default parameters. This analysis aimed to identify conserved protein domains within proteins associated with plant EVs.

### Transmembrane domain and signal peptide analyses

To predict transmembrane regions and signal peptides, the Phobius tool (Käll et al. 2004; https://phobius.sbc.su.se) was used. This software employs Hidden Markov Models (HMMs) to analyze protein sequences, enabling the simultaneous prediction of transmembrane helices and N-terminal signal peptides. All analyses were conducted using the default settings provided by the tool. For independent validation of signal peptides, SignalP 5.0 (Almagro Armenteros et al. 2019; https://services.healthtech.dtu.dk/services/SignalP-5.0/) was also employed. This analysis was performed using the default configuration, with the appropriate organism group selected based on the dataset. Both tools were applied to OGs found exclusively in gEVs, as well as those shared between gEVs and PDVs, to characterize secretion-related features and membrane associations.

### Motif discovery analysis

Motif analysis was conducted using the MEME Suite (v5.5.6; Bailey et al. 2015) in classic discovery mode, setting the maximum number of motifs to 10, a minimum motif width of 6, and an E-value threshold of ≤0.01. The analysis was performed both on the full set of identified gEV proteins and on the subset shared between gEVs and PDVs. Identified motifs were subsequently compared against the PROSITE fixed-length motif database using the Tomtom tool (Gupta et al. 2007; https://meme-suite.org/meme/tools/tomtom). Tomtom is a motif comparison algorithm that ranks target motifs based on the statistical significance of their alignment with the query motifs. For this analysis, matches with a *P*-value < 0.05 were considered significant, and motifs exhibiting the highest overlap with known motifs were selected for further consideration.

## Supplementary Material

kiag141_Supplementary_Data

## Data Availability

All relevant data can be found within the manuscript and in its supporting materials.

## Abbreviations

EVs: Extracellular vesicles
gEVs: Genuine Extracellular vesicles
PDVs: Plant-derived vesicles
ELNPs: Exosome-like nanoparticles
PDNPs: Plant-derived nanoparticles
PDENs: Plant-derived edible nanoparticles
CDD: Conserved Domain Database
NCBI: National Center for Biotechnology Information
HHMs: Hidden Markov Models
OGs: Orthogroups
dUC: Differential ultracentrifugation
IDG-UC: Iodixanol density gradient-ultracentrifugation
SDG-UC: Sucrose density gradient ultracentrifugation
SEC: Size exclusion chromatography
HSPs: Heat Shock Proteins
LEA: Late Embryogenesis Abundant
VAMPs: Vesicle-associated membrane proteins
CDPKs: Calcium-dependent kinases
GAPDH: Glyceraldehyde-3-phosphate dehydrogenase
EF1A: Elongation factor 1-alpha
TETs: Tetraspanins
STP: Sugar transport proteins
PLT: Polyol transporters
HT: Hexose transporters
MFS: Major facilitator superfamily
FLA: Fasciclin-like arabinogalactan
ENODL: Early nodulin-like
PPIases: Peptidyl-prolyl cis-trans isomerases
NLR: Nucleotide-binding leucine-rich repeat
TM: Transmembrane domains
SP: Signal peptides
RLKs: Receptor-like kinases
CBLs: Calcineurin B-like proteins
PTL: Patellin
Er: Endoplasmic Reticulum
