# Supplementary material for: Comparative proteomics reveals protein signatures shared between and unique to bona fide plant EVs and other plant-derived vesicles
Source: Plant Physiol. 2026 Mar 31;200(3):kiag141. doi: 10.1093/plphys/kiag141 (PMC13036492; doi:10.1093/plphys/kiag141)
Supplement: kiag141_Supplementary_Data [file kiag141_supplementary_data.zip › Supplementary Table 5_NEW.docx]

|  |  |  |  |
| --- | --- | --- | --- |
| **Orthogroup** | **Number of Proteins** | **Presence of TM Domain (%)** | **Proteins Exhibiting Signal Peptide (%)** |
| OG23 | 40 | 7.5% | 0% |
| OG26 | 39 | 100% | 0% |
| OG37 | 33 | 21.2% | 81.8% |
| OG45 | 30 | 30% | 83.3% |
| OG50 | 28 | 35.7% | 100% |
| OG59 | 26 | 69.2% | 92.3% |
| OG92 | 22 | 0% | 0% |
| OG115 | 19 | 0% | 84.2% |
| OG209 | 14 | 14.3% | 0% |
| OG227 | 13 | 0% | 0% |
|  |  |  |  |

**Supplementary Table S4. Overview of structural and functional characteristics of orthogroups referred to gEVs.** Composition of protein orthogroups with the number of proteins for each orthogroup, the presence/absence of transmembrane domains, and the percentage of proteins in each orthogroup that have a signal peptide. Regarding the determination of the presence or absence of transmembrane domains, the presence of at least one transmembrane domain within each orthogroup was considered. Regarding the presence of the signal peptide, it was considered as significant if the value of SP(Sec/SPI) greater than or equal to 0.7.
